# No functional differences in anatomic reconstruction with one vs. two suture anchors after non-simultaneous bilateral distal biceps brachii tendon rupture: a case report and review of the literature

**DOI:** 10.1186/s12891-020-03304-3

**Published:** 2020-04-27

**Authors:** Manuel Weißenberger, Tizian Heinz, Kilian Rueckl, Maximilian Rudert, Alexander Klug, Reinhard Hoffmann, Kay Schmidt-Horlohé

**Affiliations:** 1 Department of Orthopaedic Surgery, Koenig-Ludwig-Haus, Julius-Maximilians-University, Wuerzburg, Brettreichstr. 11, D-97074 Wuerzburg, Germany; 2 Department of Trauma and Orthopaedic Surgery, Berufsgenossenschaftliche Unfallklinik Frankfurt am Main, Frankfurt am Main, Germany; 3 Orthopaedicum Wiesbaden, Wiesbaden, Germany

**Keywords:** Non-simultaneous bilateral distal biceps tendon rupture, Distal biceps tendon repair, Anatomic reattachment, Suture anchor, Case report

## Abstract

**Background:**

Surgical reattachment of the tendon is still the gold standard for ruptures of the distal biceps brachii tendon. Several fixation techniques have been described in the literature, with suture anchors being one of the most common fixation techniques. Currently, there is no data available on how many anchors are required for a safe and stable refixation. In this case report clinical data of a patient with non-simultaneous bilateral distal biceps tendon ruptures treated with a different number of suture anchors for each side (one vs. two) are demonstrated.

**Case presentation:**

A 47-year-old factory worker suffered a rupture of the distal biceps tendon on both arms following two different occasions. The left side was fixed using a single suture anchor, while refixation on the right side was performed with two anchors.

The patient was prospectively followed for one year. Functional outcome was assessed using the Andrews Carson Score (ACS), the Oxford Elbow Score (OES), and the Disabilities of Arm, Shoulder and Hand (DASH) Score after six, twelve, 24 and 48 weeks. Furthermore, an isokinetic strength measurement for flexion strength was performed after 24 and 48 weeks.

After 48 weeks the patient presented with excellent functional outcome scores and no follow-up complications. During the follow-up period, no differences in the functional scores nor in the isokinetic flexion strength measurement could be detected. Furthermore, no radiological complications (like heterotopic ossifications) could be detected in the postoperative radiographs after one year.

**Conclusions:**

Anatomic reattachment of the distal biceps tendon is a successful operative treatment option for distal biceps tendon ruptures. Suture anchor fixation remains one of the most common techniques, as it allows fast surgery and provides good results with respect to range of motion (ROM) and functional scoring according to the current literature. However, the number of anchors required for a stable fixation remains unclear. As indicated by our presented case, we hypothesize, that there are no significant differences between a one-point or a two-point fixation. In the presented case report, no intraindividual differences between the usage of one versus two suture anchors were evident in the short-term follow-up.

## Background

Rupture of the distal biceps brachii tendon is a rare injury of the musculoskeletal system and occurs in approximately 1.2 per 100,000 persons per year, with male patients between the age of 30 and 60 years being the population at risk [1, 2]. To date, only few data exist regarding bilateral ruptures, mostly limited to case reports and small case series [3–7].

Due to the poor functional outcome when treated non-operatively, surgical reattachment of the distal biceps tendon to the bicipital tuberosity is considered the gold standard in the treatment of distal biceps tendon ruptures, especially in young and functional demanding people [8].

Currently, there is no consensus on which surgical treatment strategy might be favorable. Several different surgical approaches (one- vs. two-incision), as well as fixation techniques (suture anchors, transosseous, cortical button, etc.) are described [1, 9, 10] with lacking evidence regarding the superiority of one over the other.

Suture anchors are one of the most common fixation techniques used for anatomic reconstruction of the ruptured distal biceps tendon [11, 12]. However, the required number of suture anchors for stable fixation is still topic of ongoing discussion. In a randomized trial (Federal Ethics Committee of Hessen, Germany, study number: FF 124/2013), conducted at our institution (at the time of publication of this article still under journal’s review process), functional differences after the anatomic reattachment of ruptured distal biceps tendons using a single anchor versus two suture anchors were investigated.

Of these patients, one individual, who was treated with a single suture anchor, presented with a contralateral biceps tendon rupture during the follow-up period, and due to randomization was then treated with two suture anchors on the injured side.

This extraordinary circumstance of having a patient with bilateral biceps tendon ruptures treated differently in terms of the number of suture anchors used, provides a unique opportunity for intraindividual comparison of different treatment patterns. Clinical and functional data of this patient are demonstrated and discussed in context with current literature on anatomic reconstruction of distal biceps tendons.

## Case presentation

During the follow-up period of a randomized controlled trial, investigating functional differences by using a single suture anchor versus two suture anchors for reconstruction of acute biceps tendon ruptures, one male patient (age 47 years, factory worker, non-smoker, no comorbidities), who was initially treated with a single anchor on the left side, suffered a rupture of the contralateral biceps tendon on the right side, which was then reattached by two suture anchors following the randomization process. The contralateral distal biceps tendon rupture of the right dominant elbow occurred approximately one year after the one of the left non-dominant elbow. Both ruptures followed an acute trauma when extension load was applied to the flexed and supinated elbow by lifting up a heavy package. The mechanism of injury was identical for both ruptures and no differences between both arms in terms of peripheral vascularization, motor function and sensibility were evident at any time point. Diagnosis was easily derivable from a thorough clinical examination including the Hook-Test [13]. X-ray imaging was performed preoperatively to exclude concomitant bony lesions.

Surgery was performed under general anesthesia. A single shot of antibiotics (1 g of first-generation cephalosporine) was given preoperatively. The injured arm of the patient was positioned in supination on a radiolucent table. An anterior approach to the radial tuberosity was performed. After identification and protection of the lateral antebrachial cutaneous nerve, the radial tuberosity was detected with the forearm in full supination and decorticated for the tendon refixation. Reattachment was performed with one or two suture anchors (Arthrex, Corkscrew Suture Anchor AR-1915SNF) using biplanar fluoroscopy to verify the correct positioning. The tendon was then re-attached to the bicipital tuberosity in 60° flexion and full supination using a standard baseball-stitch suture.

Postoperatively, the patient followed our standard rehabilitation program consisting of immobilization of the elbow in a plaster cast in 90° of flexion and full supination for about one week followed by a period of six weeks with an individual adapted elbow brace allowing for active extension and passive flexion of the elbow. During this period the maximum of flexion was reduced to 30° every two weeks, starting with 90° of flexion. After six weeks, gradual biceps strengthening was applied. The patient received non-steroidal anti-inflammatory drugs for prophylaxis of heterotopic ossifications for ten days.

Functional assessment (Andrews Carson Score (ACS), Oxford Elbow Score (OES), Mayo Elbow Performance Score (MEPS), the Disabilities of Arm, Shoulder and Hand (DASH) Score) was performed preoperatively and six, twelve, 24 and 48 weeks postoperatively. In addition, isokinetic flexion strength (Isokinetik, S3, Proxomed) was measured 24 and 48 weeks postoperatively. All available radiographs were analyzed.

**Fig. 1:**
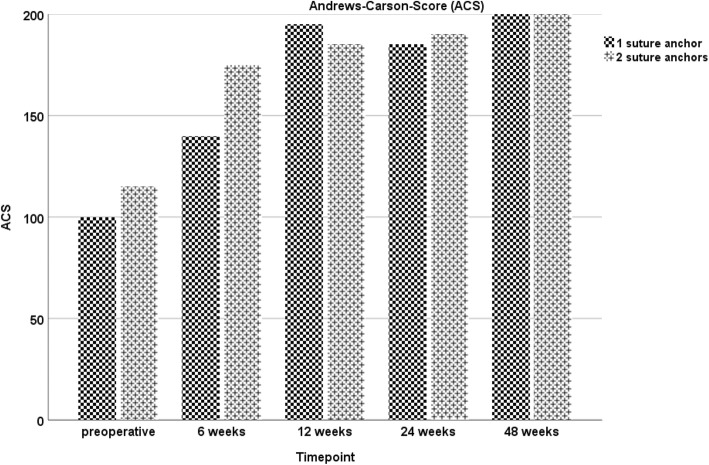
Andrews Carson Score (ACS): No significant differences in the ACS can be found at six, twelve, 24- and 48-weeks after surgery using one (left injured elbow) or two suture anchors (right injured elbow)

**Fig. 2:**
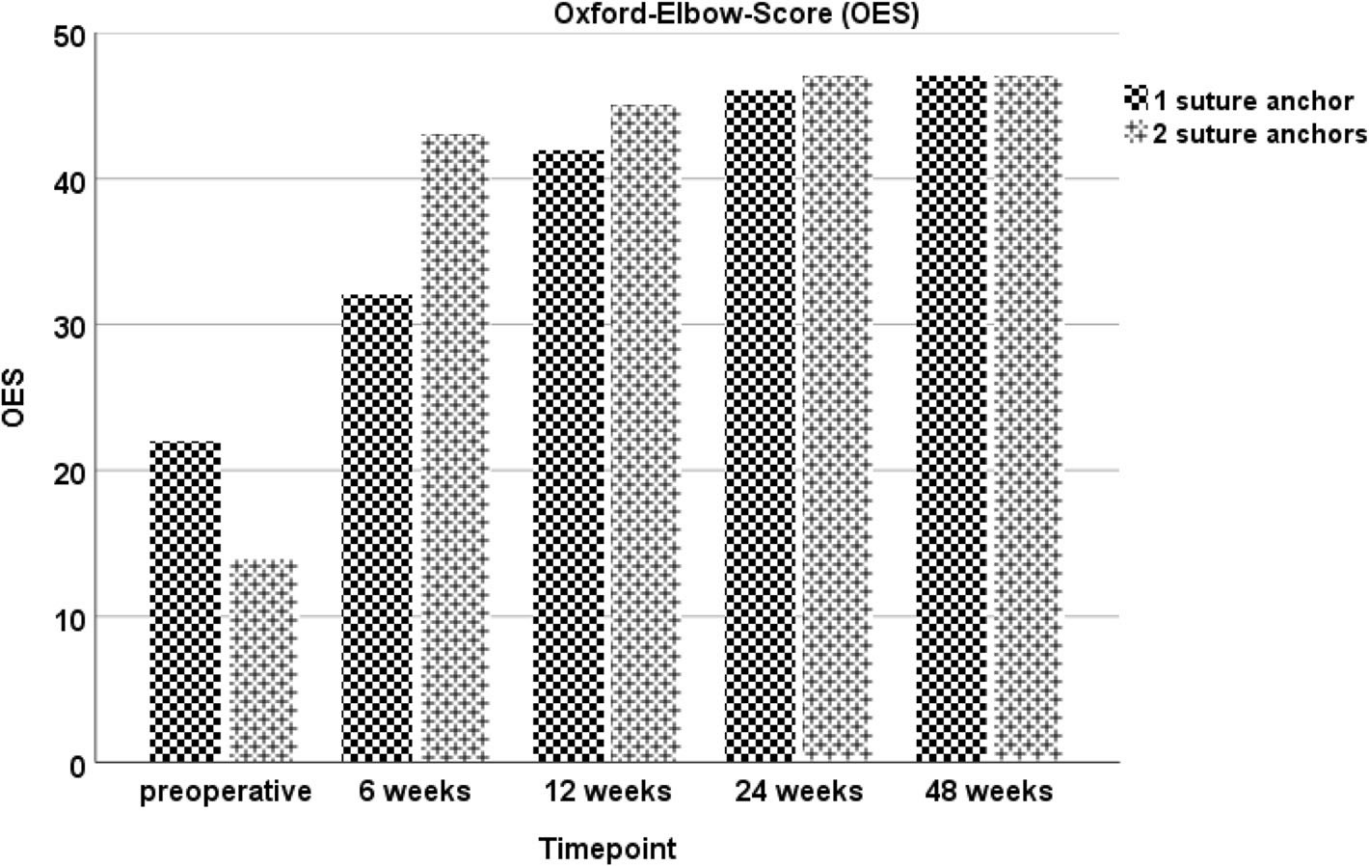
Oxford Elbow Score (OES): No significant differences in the OES can be found at six, twelve, 24- and 48-weeks following surgery for the one and two suture anchors refixation technique.

**Fig. 3:**
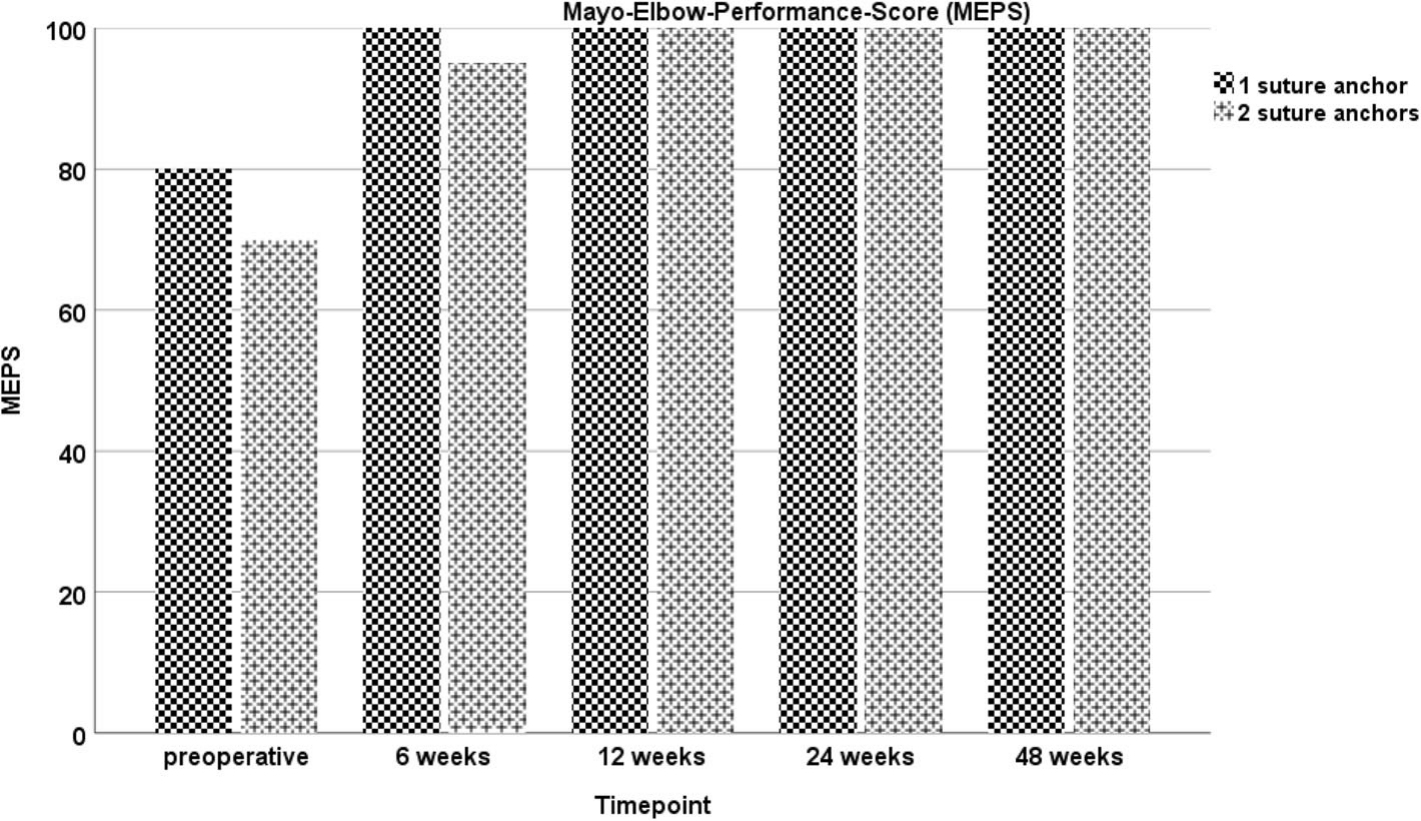
Mayo Elbow Performance Score (MEPS): No significant differences in the MEPS can be found at six, twelve, 24-, and 48-weeks following surgery for the one or two suture anchors refixation technique

**Fig. 4:**
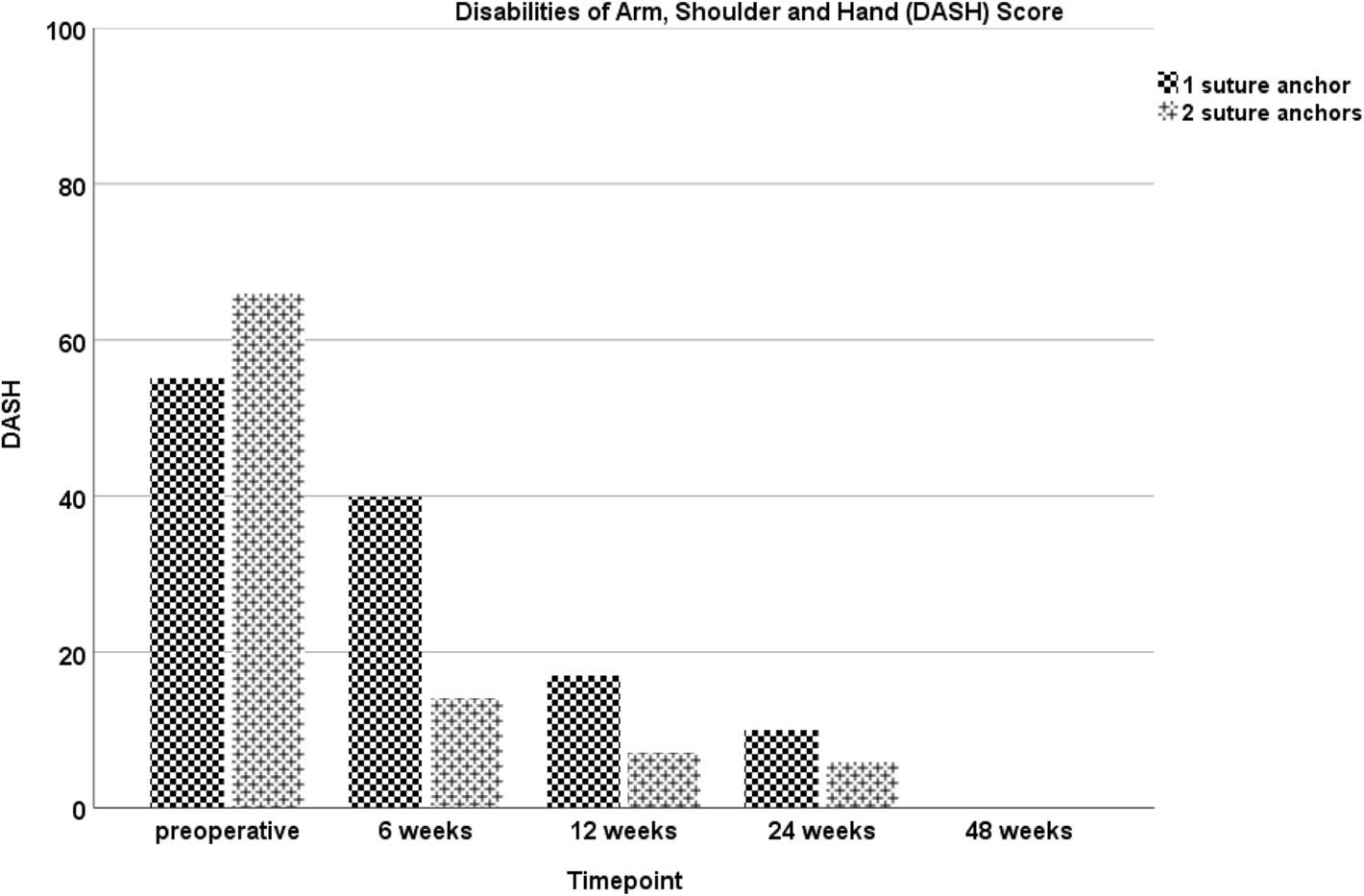
The Disabilities of Arm, Shoulder and Hand (DASH) Score: No significant differences can be found at 24- and 48-weeks following surgery for the one and two suture anchors refixation technique

**Fig. 5:**
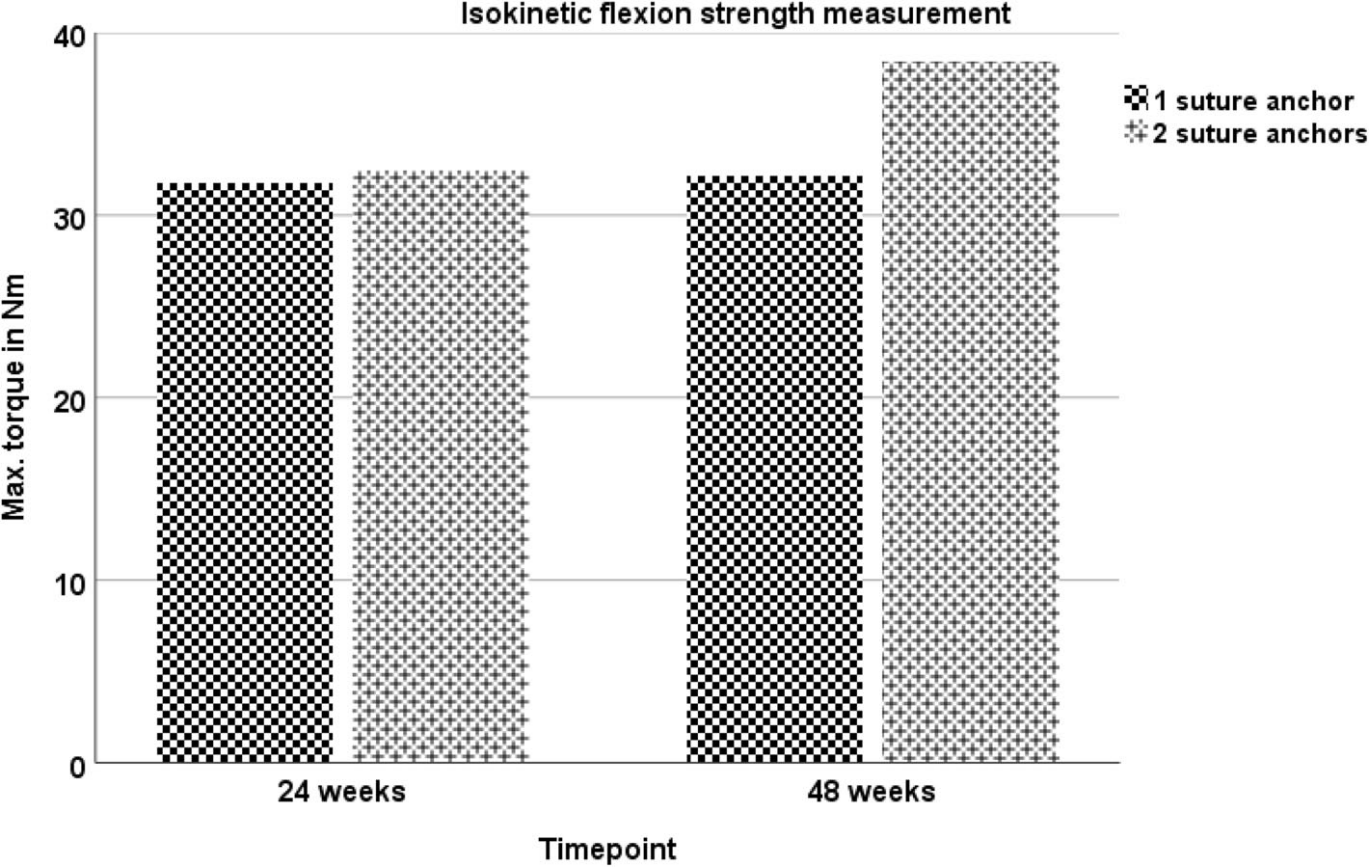
Isokinetic flexion strength measurement: There was an increase in the isokinetic flexion strength measurement both for the non-dominant left upper extremity and dominant right upper extremity. The dominant right upper extremity (2 suture anchors) showed higher values for isokinetic flexion strength 24- and 48-weeks postoperatively

During the course of the study, the patient showed an increase in all functional scores (ACS, OES, MEPS, DASH-Score), with no statistical difference between both sides at any point of follow-up evaluation. At the final visit, excellent results were assessed in the functional scores (Figs. 1, 2, 3 and 4). Furthermore, both arms showed an increase of absolute flexion strength during the study period. The isokinetic flexion strength of the non-dominant (left) arm measured 31.8 Nm at 24 weeks postoperatively and 32.2 Nm at 48 weeks postoperatively, whereas the isokinetic flexion strength of the dominant (right) arm reached 32.5 Nm at 24 weeks postoperatively and 38.4 Nm at 48 weeks postoperatively, which represents no difference between both types of fixation (Fig. 5). During the course of the follow-up, no specific surgical complications like irritation of the posterior interosseous nerve (PIN), secondary ruptures or anchor pull-outs have been recorded. Additionally, X-rays performed direct postoperatively and after one year of follow-up showed no signs of heterotopic ossifications or implant dislocations.

## Discussion and conclusion

One major finding of the presented case report is the seemingly equality of a one-point distal biceps tendon fixation over a two-point fixation in terms of functional outcome and strength measurement. However, the presented case report yields two more extraordinary aspects. Firstly, there is still quite a lack of knowledge concerning the number of suture anchors necessary for an efficient and stable refixation of ruptured distal biceps tendons, and secondly, the rareness of a non-simultaneously, bilateral distal biceps tendon rupture, whose pathogenesis, risk factors and ideal treatment is still topic of ongoing research.

In the literature the average interval between the bilateral biceps tendon ruptures varies between 2.7 and 4.6 years [3, 4, 14]. In a retrospective study [14] 23 bilateral distal biceps tendon ruptures were found in a collective of 321 patients who underwent surgical repair of the distal biceps tendon. The average interval between the bilateral tendon ruptures was 4.1 years. An average duration of 2.7 and 4.6 years till the onset of the acute contralateral tendon rupture was shown by several studies [3, 4]. Several risk factors for bilateral tendon ruptures have also been reported in the literature. Therefore, end-stage renal diseases, hyperparathyroidism as well as the use of steroids or quinolone antibiotics were found being associated with an increased risk of biceps tendon ruptures [15]. Weightlifters and bodybuilders have also been reported to be at higher risk for bilateral tendon injuries [6, 7]. The use of anabolic steroids is also discussed as a further risk factor [4, 7, 15]. Although, several risk factors for unilateral distal biceps tendon ruptures are discussed in the literature [1], no specific risk factors for bilateral distal biceps tendon ruptures are described in the literature until now.

There exist different theories trying to explain the etiology of distal biceps tendon rupture. There are described two stages in tearing of the distal biceps tendon [16]. Incomplete tears because of pathological changes in the tendon represent the first stage. The second stage is finally a complete rupture of the distal biceps tendon after the lacertus fibrosus tears during a muscle contraction [16].

Furthermore, it seems that a certain hypovascular zone and a mechanical impingement during forearm rotation can contribute to a rupture of the distal biceps tendon [17]. And even degenerative changes in ruptured distal biceps tendons can be found in microscopic evaluation [18]. Some authors support the theory that a systemic etiology, chronic tendinitis or anatomic variants can contribute to bilateral ruptures of the distal biceps tendon in a single individual [14].

To date, several surgical procedures are described for reattachment of distal biceps tendons. The differences center around the number of incisions, the site of tendon attachment, the type of fixation device, and the use of grafts when chronic detachment is encountered. Originally, the biceps tendon was reattached through an anterior incision [19], but the number of reported neurovascular complications is concerning [20], which is why some authors favor a double-incision approach. According to Kodde et al. [20] the double-incision approach had significantly fewer complications than the single-incision anterior approach in their latest systematic review of 40 studies. However, the clinical outcome remains controversial [21, 22], as some authors reported a significantly greater proportion of unsatisfactory results than with single-incision repair [23]. In the only randomized controlled trial to this topic, no significant differences in outcomes between the single- and double-incision distal biceps repair techniques other than a 10% advantage in final flexion strength with the latter could be shown [9]. But not only the surgical approach remains topic of ongoing discussion, but also the optimal refixation technique. During the last decade numerous products were introduced on the market, of which most of them can be assigned to one of the following kinds of fixation strategy: suture anchors, interference screws, or cortical buttons. The different fixation techniques have already been compared in some biomechanical studies [10, 24–26], most of them demonstrating a significantly higher load to failure for cortical buttons compared to bone tunnels, suture anchors, and interference screws. Despite the biomechanical superiority, literature indicates, that comparable good to excellent results are possible with all of these fixation techniques [2, 11, 27], although statistical evidence and RCTs are still lacking. Suture anchor fixation remains one of the most common techniques, as it allows fast surgery by a single-incision approach and provides high patient’s satisfaction and good results with respect to ROM and functional scoring [12, 28]. However, the number of anchors required for a stable fixation remains unclear, as most studies use two or even more anchors for tendon refixation [12, 28, 29], which is the reason, why we run a randomized controlled trial comparing the outcomes of a one-point vs. a two-point fixation. As indicated by our presented case, we hypothesize, that there are no significant differences between both procedures. Data of our soon going to be published RCT are also suggestive for that hypothesis. This case report of a 47-year-old man showed no intraindividual difference in terms of the functional scores and isokinetic measurements of flexion strength between using one or two suture anchors. As the usage of a single suture anchor for distal biceps tendon repair is associated with less implants in vivo and economic benefits while providing identical clinical results, authors highly favor the usage of a single suture anchor over two or even more anchors. However, the presented data should be considered as short-term follow-up data. Although most biomechanical studies examine the pull-out strength of two suture anchors or a two-point fixation principle, the clinical relevance of this issue has not been investigated, yet. In our opinion, a one-point fixation might provide enough tendon healing in vivo, if the rehabilitation protocol is adequate. Therefore, further studies on this topic will be necessary before a final treatment recommendation can be made.

Summarizing, the usage of one suture anchor for distal biceps tendon refixation seems to provide a safe and stable fixation technique with both patient-related and economic benefits and showed in this presented case no disadvantages concerning the functional outcome and flexion strength.

## Data Availability

The datasets used and/or analyzed during the current study are available from the corresponding author on reasonable request.

## Abbreviations

ACS: Andrews Carson Score
OES: Oxford Elbow Score
DASH-Score: Disabilities of Arm, Shoulder and Hand Score
ROM: Range of motion
MEPS: Mayo Elbow Performance-Score
PIN: Posterior interosseous nerve
